# Report of a Case of Phlegmon Starting as a Peritonsillar Abscess and Extending Downward as Far as the Second Ring of the Trachea

**Published:** 1913-09

**Authors:** George L. Richards

**Affiliations:** Fall River


### Report of a Case of Phlegmon Starting as a Peritonsillar Abscess and Extending Downward as Far as the Second Ring of the Trachea.

This case had its origin primarily in a peritonsillar abscess and secondarily in a diseased tonsil, and is a good example of a severe phlegmon.—Dr. George L. Richards, Fall River.

